# Deformation investigation of electromagnetic diaphragm pump rubber diaphragm

**DOI:** 10.1371/journal.pone.0304052

**Published:** 2024-06-24

**Authors:** Yu Liao, Heng Wang, Biao Chen, Yinshui Liu

**Affiliations:** 1 School of Mechanical Science and Engineering, Huazhong University of Science and Technology, Wuhan, China; 2 Shentuo (Beijing) Science & Technology Co., Ltd, Beijing, China; 3 Midea Group, Foshan, China; Ningbo University, CHINA

## Abstract

A diaphragm pump is a type of volumetric pump that has excellent sealing performance. An electromagnetic diaphragm pump is a kind of widely adopted diaphragm pump that has a simple structure, low power loss, and high cost performance. However, the calculation method of deformation for the electromagnetic diaphragm pump rubber diaphragm is presently lacking. Herein, a calculating method of deformation for the electromagnetic diaphragm pump rubber diaphragm is proposed. By establishing and analyzing a deformation model of the electromagnetic diaphragm pump rubber diaphragm, a theoretical relationship between the deformation of the electromagnetic diaphragm pump rubber diaphragm, the size of the electromagnetic diaphragm pump rubber diaphragm and the pressure of fluid is determined. The experimental results indicate that the biggest difference between the tested axial deformation and the calculated axial deformation of the electromagnetic diaphragm pump rubber diaphragm is 0.04 mm and the calculation results show agreement with the experimental results.

## Section 1: Introduction

A diaphragm pump is a kind of volumetric pump that has promising sealing performance, and has been widely employed in petroleum, medicine industries, biological research and selective catalytic reduction (SCR) systems [1–4]. The past decade has seen rapid advances in the diaphragm pump [5–8]. As a significant type of the diaphragm pump, an electromagnetic diaphragm pump has simple structure, low power loss, and high cost performance.

However, the calculation method of deformation for the electromagnetic diaphragm pump rubber diaphragm is presently lacking.

Numerous researchers have proposed various methods to analyze the deformation of diaphragm pumps [9–13]. W Yang and S Gu [14] established a finite element model of titanium diaphragm used in a spacecraft propellant tank by using MSC. Marc software to study deformation feature of the diaphragm during the reversal process for spacecraft. The simulation results imply that the deformation for the diaphragm is steady in the reversal process of the spacecraft. G Gerard and P Rapirno [15] assumed that the curve formed after deformation is a parabola according to the deformation feature of the diaphragm in the motor-driven diaphragm pump, and determined the basic parameters of parabola equation according to the test results, calculated the radial curvature radius, circumferential curvature radius and stress distribution of the diaphragm according to parabola equation. CG Wang and YQ You [16] established a simplified three-dimensional (3D) numerical model of the diaphragm in the micro diaphragm pump, used the finite element analysis software ANSYS to carry out structural nonlinear analysis and fluid structure coupling transient analysis, and optimized the size of the diaphragm according to the stress distribution. XW Pan and SD Yang [17] established a transient three-dimensional fluid-structure interaction model to investigate the deformation of a diaphragm pump used in exhaust gas treatment system. The simulation results imply that the deformation is only determined by the back pressure of the diaphragm pump. Samridhi and M Kumar [18] used finite element method to evaluate stress of silicon diaphragm at different frequencies. However, these researchers have only analyzed the deformation of the motor-driven diaphragm pump, and have not calculated the deformation of the electromagnetic diaphragm pump rubber diaphragm.

In this study, a calculating method of the deformation for the electromagnetic diaphragm pump rubber diaphragm is proposed. A theoretical relationship between the deformation of the electromagnetic diaphragm pump rubber diaphragm, the size of the electromagnetic diaphragm pump rubber diaphragm and the pressure of fluid is ascertained by establishing and analyzing the deformation model of the electromagnetic diaphragm pump rubber diaphragm. The experimental results reveal that the biggest difference between the tested axial deformation and the calculated axial deformation of the electromagnetic diaphragm pump rubber diaphragm is 0.04 mm and the calculation results show agreement with the experimental results. This research may provide theoretical guidance for the design of high-cost-performance and simple structure electromagnetic diaphragm pump rubber diaphragm.

The rest of this paper is organized as follows: structure and working philosophy of the electromagnetic diaphragm pump are demonstrated in Section 2. Deformation analysis of the electromagnetic diaphragm pump rubber diaphragm is given in Section 3. Analysis on effective displacement of electromagnetic diaphragm pump is illustrated in Section 4. Experimental results are presented in Section 5. Finally, Section 6 concludes the work.

## Section 2: Structure and working philosophy of electromagnetic diaphragm pump

**Fig 1:**
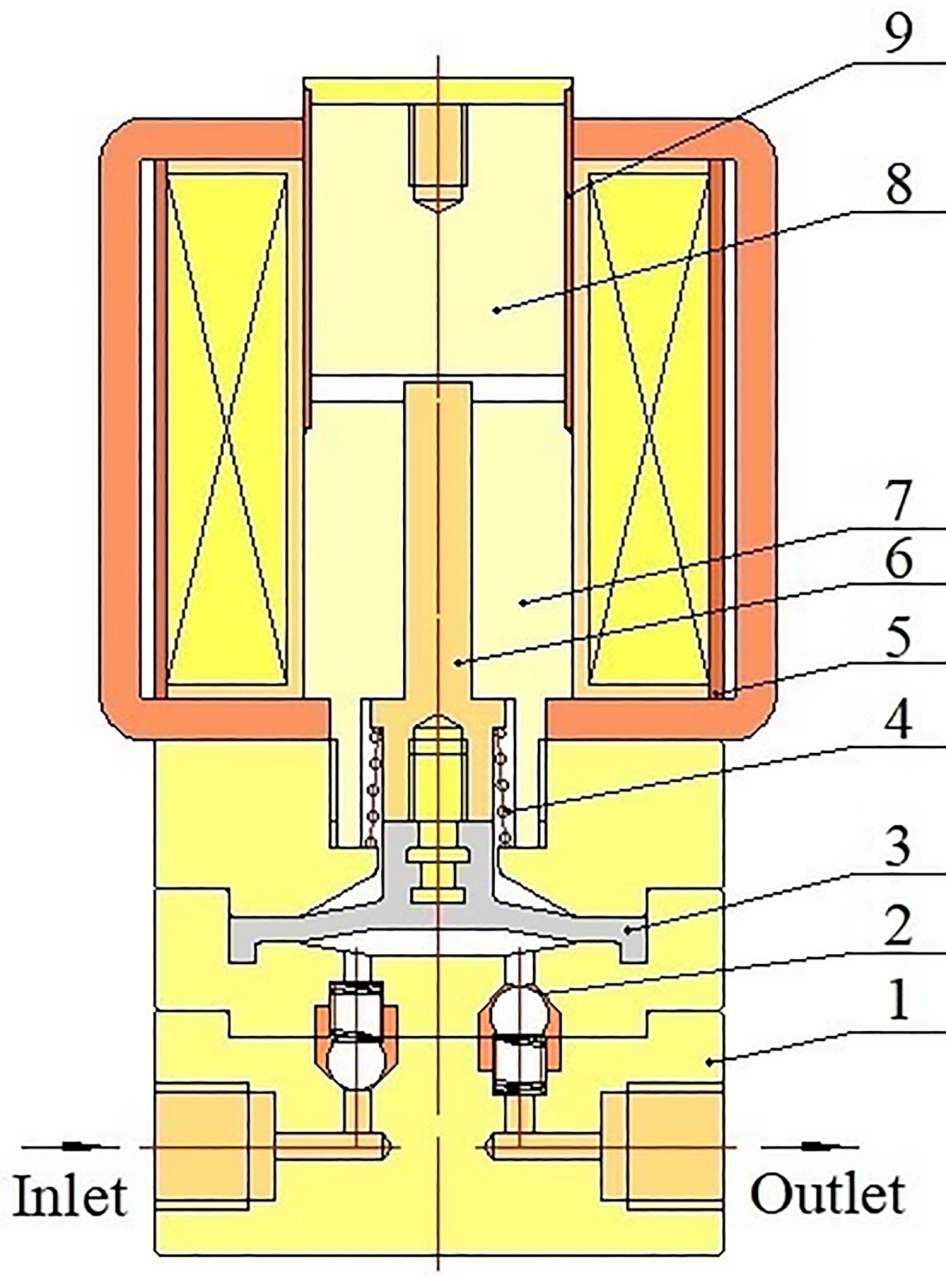
Structural schematic of electromagnetic diaphragm pump: 1-pump body; 2-single-direction valve; 3-electromagnetic diaphragm pump rubber diaphragm; 4-spring; 5-electromagnetic coil; 6-ejector pin; 7-iron core; 8-armature; 9-sleeve.

The electromagnetic diaphragm pump is mainly composed of electromagnetic coil, iron core, ejector pin, armature, sleeve, spring, pump body, electromagnetic diaphragm pump rubber diaphragm and single-direction valves, as depicted in Fig 1. The electromagnetic diaphragm pump rubber diaphragm is directly connected with the iron core, and the electromagnetic diaphragm pump rubber diaphragm and the pump body constitute a working cavity that has variable volume.

The working philosophy of the electromagnetic diaphragm pump is: when the electromagnetic coil is not energized, the armature drives the ejector pin and the electromagnetic diaphragm pump rubber diaphragm to move upwards under the action of spring force, then the volume of the working cavity increases and the pressure of fluid in the working cavity decreases. The single-direction valve near the inlet opens when the pressure of fluid in the working cavity is lower than the pressure of fluid in the inlet, and fluid is inhaled into the working cavity. When the electromagnetic coil is energized, the armature is attracted by the electromagnetic force to drive the ejector pin and the electromagnetic diaphragm pump rubber diaphragm downward, then the volume of the working cavity drops and the pressure of fluid in the working cavity increases. When the pressure of fluid in the working cavity is higher than the pressure of fluid in the outlet, the single-direction valve beside the outlet opens, and fluid is discharged out of the working cavity. The electromagnetic diaphragm pump is driven by pulse frequency modulation (PFM) signal to repeatedly inhale and discharge fluid.

## Section 3: Deformation analysis of electromagnetic diaphragm pump rubber diaphragm

**Fig 2:**
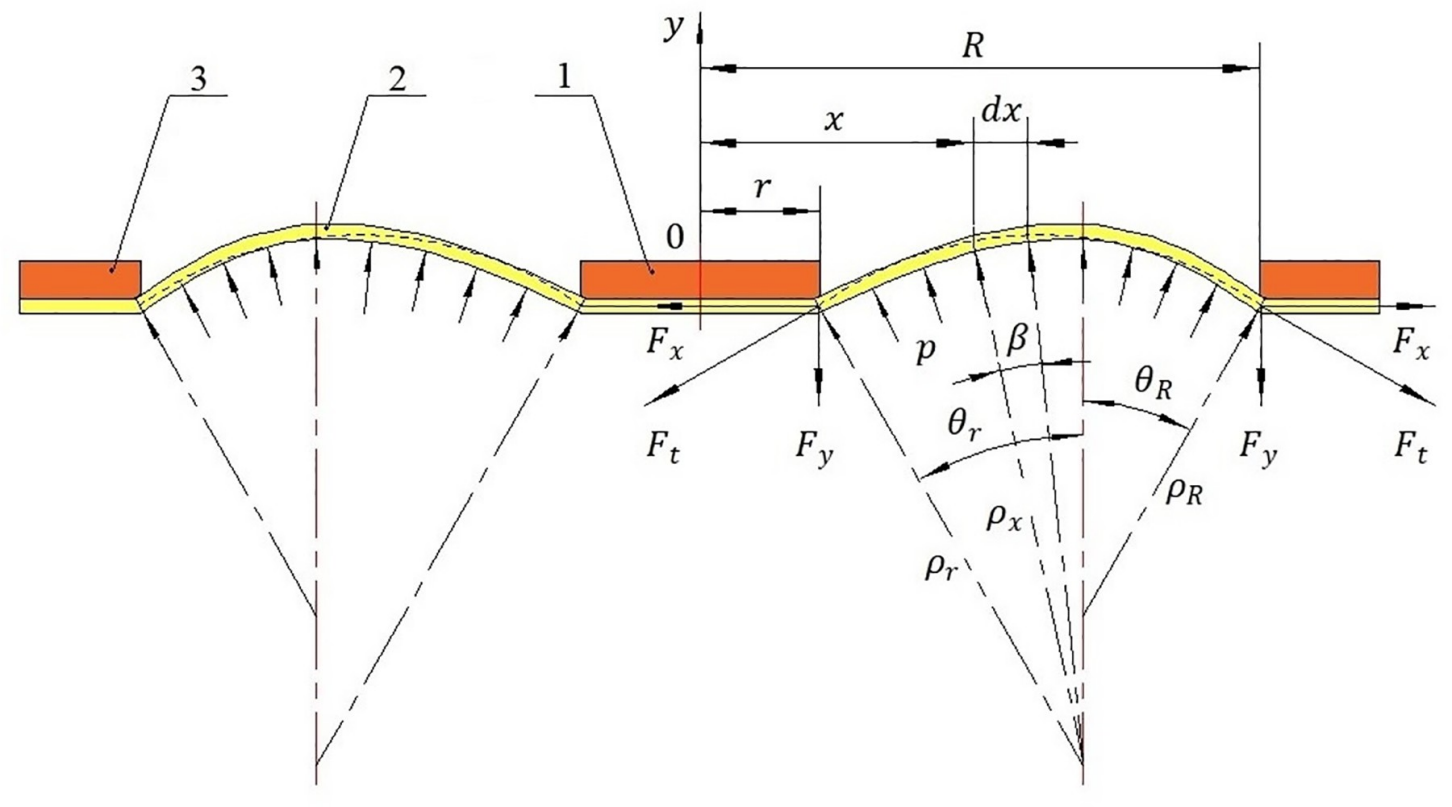
Deformation model of electromagnetic diaphragm pump rubber diaphragm: 1-the inner driving ring; 2-electromagnetic diaphragm pump rubber diaphragm; 3-the outer fixing ring.

According to the traditional design theory of diaphragms, in the process of diaphragms motion, the deformation of diaphragms consists of two parts: bending and stretching. Because the thickness of the electromagnetic diaphragm pump rubber diaphragm is tiny, the theory of elastic thin shell that ignores the bending moment can be used in the limited deformation range of the electromagnetic diaphragm pump rubber diaphragm, that is, the internal force generated by bending deformation can be ignored, and only the tangential stress generated by tensile deformation is considered. Based on the above theories, a deformation model of the electromagnetic diaphragm pump rubber diaphragm is established to calculate the deformation of the electromagnetic diaphragm pump rubber diaphragm, which is manifested in Fig 2. If the tangential stress generated by tensile deformation is ignored, the omission of neglecting the internal force generated by bending deformation may lead to significant errors in the deformation analysis.

As depicted in Fig 2, *r* is the radius for the inner driving ring of the electromagnetic diaphragm pump rubber diaphragm and *R* is the radius for the outer fixing ring of the electromagnetic diaphragm pump rubber diaphragm. Besides, it can be found in Fig 2 that the coordinate for the center of the inner driving ring is (0, 0). In the annular deformation region of the electromagnetic diaphragm pump rubber diaphragm from *r* to *R*, the tangential tension at any position *x* along the radial direction of the electromagnetic diaphragm pump rubber diaphragm is calculated as

$$F_{t}=2\pi xt\sigma_{x}=2\pi xtE\varepsilon_{x}$$ (1)

where *r*≤ *x* ≤ *R* is a variable, *t* is the thickness of the electromagnetic diaphragm pump rubber diaphragm, *σ*_*x*_ is the tangential stress at any position *x* along the radial direction of the electromagnetic diaphragm pump rubber diaphragm, *E* and *ε*_x_ are the elastic modulus of the electromagnetic diaphragm pump rubber diaphragm and the tangential strain at any position *x* along the radial direction of the electromagnetic diaphragm pump rubber diaphragm, respectively. If the electromagnetic diaphragm pump rubber diaphragm is sandwiched with braid, then *E* is the elastic modulus of the braid. In a steady state, the tangential tensions at any position *x* along the radial direction of the electromagnetic diaphragm pump rubber diaphragm are the same, and the stress in the annular electromagnetic diaphragm pump rubber diaphragm is larger than the stress outside the annular electromagnetic diaphragm pump rubber diaphragm. The tangential stress at any position *x* along the radial direction of the electromagnetic diaphragm pump rubber diaphragm can be written as

$$\sigma_{x}=\frac{r\sigma_{r}}{x}=\frac{R\sigma_{R}}{x}$$ (2)

The tangential strain at any position *x* along the radial direction of the electromagnetic diaphragm pump rubber diaphragm is expressed as

$$\varepsilon_{x}=\frac{r\varepsilon_{r}}{x}=\frac{R\varepsilon_{R}}{x}$$ (3)

By taking advantage of (3), the average tangential strain of the deformed electromagnetic diaphragm pump rubber diaphragm is expressed as

$$\varepsilon_{a}=\frac{\varepsilon_{r}+\varepsilon_{R}}{2}=\frac{\varepsilon_{r}+\frac{r}{R}\varepsilon_{r}}{2}=\frac{(R+r)\varepsilon_{r}}{2R}$$ (4)

Take a small variable d*x* at any position *x* in annular deformation area of the electromagnetic diaphragm pump rubber diaphragm, *β* is the angle that is corresponding to d*x* on the electromagnetic diaphragm pump rubber diaphragm. In the arc section that the angle *β* is opposite to, the normal force that results from the pressure of fluid is obtained.

$$F_{r}=2\pi xp\beta \rho_{x}$$ (5)

where *ρ*_x_ is the curvature radius of the deformed electromagnetic diaphragm pump rubber diaphragm arc at any position *x* and *p* is the pressure of fluid. Within the angle *β*, the normal force that results from the pressure of fluid and the tangential tension at any position *x* along the radial direction of the electromagnetic diaphragm pump rubber diaphragm satisfy

$$F_{r}=2F_{t}\sin\frac{\beta }{2}$$ (6)

When the angle *β* is overwhelmingly small, the normal force that results from the pressure of fluid and the tangential tension at any position *x* along the radial direction of the electromagnetic diaphragm pump rubber diaphragm satisfy

$$F_{r}={\beta F}_{t}$$ (7)

By combining (1), (3), (5) and (7), the following result can be derived.

$$\rho_{x}=\frac{t\sigma_{x}}{p}=\frac{tr\sigma_{r}}{px}=\frac{trE\varepsilon_{r}}{px}$$ (8)

According to (8), the curvature radius of the deformed electromagnetic diaphragm pump rubber diaphragm arc at position *r* can be computed as

$$\rho_{r}=\frac{t\sigma_{r}}{p}=\frac{tE\varepsilon_{r}}{p}$$ (9)

Moreover, the curvature radius of the deformed electromagnetic diaphragm pump rubber diaphragm arc at position *R* is expressed as

$$\rho_{R}=\frac{t\sigma_{R}}{p}=\frac{tEr\varepsilon_{r}}{pR}$$ (10)

By taking advantage of (9) and (10), the average curvature radius of the deformed electromagnetic diaphragm pump rubber diaphragm arc is calculated as

$$\rho_{a}=\frac{\rho_{r}+\rho_{R}}{2}=\frac{tE(R+r)\varepsilon_{r}}{2pR}$$ (11)

At the fixed position *x* = *r* of the inner driving ring and the fixed position *x* = *R* of the outer fixing ring, the horizontal component forces that the electromagnetic diaphragm pump rubber diaphragm bears satisfy

$$F_{t}{\cos\theta }_{r}=F_{t}{cos\theta }_{R}$$ (12)

where *θ*_r_ is the included angle between tangent line for the inner driving ring of the electromagnetic diaphragm pump rubber diaphragm and *x*-axis, *θ*_R_ is the included angle between tangent line for the outer fixing ring of the electromagnetic diaphragm pump rubber diaphragm and *x*-axis. According to (12), the following result is obtained.

$$\theta_{r}=\theta_{R}$$ (13)

At the fixed position *r* of the inner driving ring and the fixed position *R* of the outer fixing ring, the sum of component forces that the electromagnetic diaphragm pump rubber diaphragm bears in the vertical direction is equal to the force that results from the pressure of fluid, namely:

$$F_{t}{\sin\theta }_{r}+F_{t}{\sin\theta }_{R}=2F_{t}{sin\theta }_{r}=\pi (R^{2}-r^{2})p$$ (14)

A deformation function of the electromagnetic diaphragm pump rubber diaphragm *y* is built to calculate the deformation, and the curvature radius for the deformation function of the electromagnetic diaphragm pump rubber diaphragm is supposed to be equal to the curvature radius of the deformed electromagnetic diaphragm pump rubber diaphragm arc at any position *x*. The following result can be derived from the formula of curvature radius and (8).

$$\frac{{\left[1+{\left(y^{\prime }\right)}^{2}\right]}^{\frac{3}{2}}}{y^{"}}=\rho_{x}=\frac{trE\varepsilon_{r}}{px}$$ (15)

By taking advantage of (15), the following result is can be obtained.

$$\frac{rtE\varepsilon_{r}}{p}\frac{dy^{\prime }}{{\left[1+{\left(y^{\prime }\right)}^{2}\right]}^{\frac{3}{2}}}=xdx$$ (16)

According to (16), the derivative for the deformation function of the electromagnetic diaphragm pump rubber diaphragm is determined.

$$y^{\prime }=-\frac{x^{2}+a}{\sqrt{{\left(\frac{2rtE\varepsilon_{r}}{p}\right)}^{2}-{\left(x^{2}+a\right)}^{2}}}$$ (17)

where *a* is a constant. According to (13), the value of *a* is computed as

$$a=-\frac{R^{2}{+r}^{2}}{2}$$ (18)

By substituting (18) into (17), the derivative for the deformation function of the electromagnetic diaphragm pump rubber diaphragm is described as

$$y^{\prime }=-\frac{x^{2}-\frac{R^{2}{+r}^{2}}{2}}{\sqrt{{\left(\frac{2rtE\varepsilon_{r}}{p}\right)}^{2}-{\left(x^{2}-\frac{R^{2}{+r}^{2}}{2}\right)}^{2}}}$$ (19)

When *y*′ = 0, the value of *x* is described by

$$R_{0}=\sqrt{\frac{R^{2}{+r}^{2}}{2}}$$ (20)

where *R*_0_ is the radius of the dividing surface between the pressure acting on the inner driving ring of the electromagnetic diaphragm pump rubber diaphragm that results from the fluid and the pressure acting on the outer fixing ring of the electromagnetic diaphragm pump rubber diaphragm that results from the fluid. According to (20), the inner driving ring and the outer fixing ring of the electromagnetic diaphragm pump rubber diaphragm bear the same vertical force that results from the pressure of fluid, namely:

$$F_{1}=\pi (R_{0}^{2}-r^{2})p=F_{2}=\pi (R^{2}-R_{0}^{2})p=\frac{\pi (R^{2}-r^{2})p}{2}$$ (21)

It is obvious that (19) cannot be solved using the integral method. To analyze the deformation of the electromagnetic diaphragm pump rubber diaphragm, a deforming curve function of the electromagnetic diaphragm pump rubber diaphragm *f*(*x*) is constructed. The deforming curve function of the electromagnetic diaphragm pump rubber diaphragm is assumed to be an arc, the radius for the deforming curve function of the electromagnetic diaphragm pump rubber diaphragm is presumed to be equal to the average curvature radius of the deformed electromagnetic diaphragm pump rubber diaphragm arc, and the length for the deforming curve function of the electromagnetic diaphragm pump rubber diaphragm from *r* to *R* is supposed to be equal to the length of the deformed electromagnetic diaphragm pump rubber diaphragm arc. It can be found in Fig 2 that the coordinates for intersection points of the inner driving ring and *x*-axis include (*r*, 0) and (-*r*, 0), and the coordinates for intersection points of the outer fixing ring and *x*-axis include (*R*, 0) and (-*R*, 0). By taking advantage of (11), the circle equation of the deformed electromagnetic diaphragm pump rubber diaphragm constructed by coordinates (*r*, 0), (*R*, 0) and the average curvature radius of the deformed electromagnetic diaphragm pump rubber diaphragm arc is calculated as

$${\left(x-\frac{R+r}{2}\right)}^{2}+{\left\{f(x)+\sqrt{{\left[\frac{tE(R+r)\varepsilon_{r}}{2pR}\right]}^{2}-{\left(\frac{R-r}{2}\right)}^{2}}\right\}}^{2}={\left[\frac{tE(R+r)\varepsilon_{r}}{2pR}\right]}^{2}$$ (22)

The deforming curve function of the electromagnetic diaphragm pump rubber diaphragm is expressed as

$$f(x)=\sqrt{{\left[\frac{tE(R+r)\varepsilon_{r}}{2pR}\right]}^{2}-{\left(x-\frac{R+r}{2}\right)}^{2}}-\sqrt{{\left[\frac{tE(R+r)\varepsilon_{r}}{2pR}\right]}^{2}-{\left(\frac{R-r}{2}\right)}^{2}}$$ (23)

According to (23), the derivative for the deforming curve function of the electromagnetic diaphragm pump rubber diaphragm is described by

$$f^{\prime }(x)=-\frac{x-\frac{R+r}{2}}{\sqrt{{\left(\frac{tE(R+r)\varepsilon_{r}}{2pR}\right)}^{2}-{\left(x-\frac{R+r}{2}\right)}^{2}}}$$ (24)

By taking advantage of (23), the length for the deforming curve function of the electromagnetic diaphragm pump rubber diaphragm from *r* to *R* is described as

$$L_{f}=\int_{r}^{R}\sqrt{1+{\left[\frac{df(x)}{dx}\right]}^{2}}dx=\int_{r}^{R}\frac{\frac{tE(R+r)\varepsilon_{r}}{2pR}}{\sqrt{{\left[\frac{tE(R+r)\varepsilon_{r}}{2pR}\right]}^{2}-{\left(x-\frac{R+r}{2}\right)}^{2}}}dx=\frac{tE(R+r)\varepsilon_{r}}{pR}\arcsin\frac{pR(R-r)}{tE(R+r)\varepsilon_{r}}$$ (25)

Expanding $\arcsin\frac{pR(R-r)}{tE(R+r)\varepsilon_{r}}$ by power series and taking the first two terms, the length for the deforming curve function of the electromagnetic diaphragm pump rubber diaphragm *r* to *R* is calculated as

$$L_{f}=R-r+\frac{p^{2}R^{2}(R-r{)}^{3}}{6(R+r{)}^{2}t^{2}E^{2}\varepsilon_{r}^{2}}$$ (26)

Table 1 demonstrates the relative error between the first two terms of power series expansion and (25) changing with $\arcsin\frac{pR(R-r)}{tE(R+r)\varepsilon_{r}}$. It can be found in Table 1 that the relative error between the first two terms of power series expansion and (25) decreases by decreasing $\arcsin\frac{pR(R-r)}{tE(R+r)\varepsilon_{r}}$. Because the rated pressure of the electromagnetic diaphragm pump is 0.3 MPa and $\arcsin\frac{pR(R-r)}{tE(R+r)\varepsilon_{r}}$ is tiny. As a result, the relative error between the first two terms of power series expansion and (25) is quite small.

**Table 1:** The effect of $\arcsin\frac{pR(R-r)}{tE(R+r)\varepsilon_{r}}$ on the relative error between the first two terms of power series expansion and (25).

$\arcsin\frac{pR(R-r)}{tE(R+r)\varepsilon_{r}}$	The relative error between the first two terms of power series expansion and (25)
$\frac{\pi }{2}$	25.7%
$\frac{\pi }{3}$	7.2%
$\frac{\pi }{6}$	3.5%

According to the theory of elastic deformation, the length of the deformed electromagnetic diaphragm pump rubber diaphragm arc is given by

$$L_{d}=(R-r)(1+\varepsilon_{a})$$ (27)

By substituting (4) into (27), the length of the deformed electromagnetic diaphragm pump rubber diaphragm arc is obtained.

$$L_{d}=(R-r)\left[1+\frac{(R+r)\varepsilon_{r}}{2R}\right]$$ (28)

The tangential strain at position *r* along the radial direction of the electromagnetic diaphragm pump rubber diaphragm can be obtained by combining (26) and (28).

$$\varepsilon_{r}=\sqrt[{3}]{\frac{p^{2}R^{3}(R-r{)}^{2}}{3(R+r{)}^{3}t^{2}E^{2}}}$$ (29)

The tangential stress at position *r* along the radial direction of the electromagnetic diaphragm pump rubber diaphragm is written as

$$\sigma_{r}=\varepsilon_{r}E=\sqrt[{3}]{\frac{p^{2}R^{3}(R-r{)}^{2}E}{3(R+r{)}^{3}t^{2}}}$$ (30)

The tangential strain at position *R* along the radial direction of the electromagnetic diaphragm pump rubber diaphragm can be given by

$$\varepsilon_{R}=\frac{r\varepsilon_{r}}{R}=\sqrt[{3}]{\frac{p^{2}r^{3}(R-r{)}^{2}}{3(R+r{)}^{3}t^{2}E^{2}}}$$ (31)

According to (31), the tangential stress at position *R* along the radial direction of the electromagnetic diaphragm pump rubber diaphragm can be presented by

$$\sigma_{R}=\varepsilon_{R}E=\sqrt[{3}]{\frac{p^{2}r^{3}(R-r{)}^{2}E}{3(R+r{)}^{3}t^{2}}}$$ (32)

By taking advantage of (29), the tangential strain at any position *x* along the radial direction of the electromagnetic diaphragm pump rubber diaphragm is described by

$$\varepsilon_{x}=\frac{r\varepsilon_{r}}{x}=\frac{1}{x}\sqrt[{3}]{\frac{p^{2}r^{3}R^{3}(R-r{)}^{2}}{3(R+r{)}^{3}t^{2}E^{2}}}$$ (33)

In addition, the tangential stress at any position *x* along the radial direction of the electromagnetic diaphragm pump rubber diaphragm can be determined by taking advantage of (2) and (33).

$$\sigma_{x}=\varepsilon_{x}E=\frac{1}{x}\sqrt[{3}]{\frac{p^{2}r^{3}R^{3}(R-r{)}^{2}E}{3(R+r{)}^{3}t^{2}}}$$ (34)

It can be seen from (34) that the tangential stress at any position *x* along the radial direction of the electromagnetic diaphragm pump rubber diaphragm is directly proportional to the radius for the inner driving ring of the electromagnetic diaphragm pump rubber diaphragm and the radius for the outer fixing ring, increases by increasing the pressure of fluid and the elastic modulus of the electromagnetic diaphragm pump rubber diaphragm, and decreases by increasing the thickness of the electromagnetic diaphragm pump rubber diaphragm. For an electromagnetic diaphragm pump rubber diaphragm that has constant thickness, maximum stress appears in the inner driving ring of the electromagnetic diaphragm pump rubber diaphragm, and minimum stress appears in the outer fixing ring of the electromagnetic diaphragm pump rubber diaphragm, and the ratio of the former to the latter is $\frac{R}{r}$. If the thickness of the electromagnetic diaphragm pump rubber diaphragm can vary, and the thickness of the electromagnetic diaphragm pump rubber diaphragm at any position *x* satisfies $t_{x}=\frac{rt_{r}}{x}$, then the tangential stresses acting on the inner driving ring of the electromagnetic diaphragm pump rubber diaphragm and the outer fixing ring are the same, namely:

$$\sigma_{x}=\sigma_{r}=\sigma_{R}=\frac{pR}{t_{r}}$$ (35)

By substituting (29) into (23), the deforming curve function of the electromagnetic diaphragm pump rubber diaphragm is described as

$$f(x)=\sqrt{{\left[\frac{tE(R-r{)}^{2}}{24p}\right]}^{\frac{2}{3}}-{\left(x-\frac{R+r}{2}\right)}^{2}}-\sqrt{{\left[\frac{tE(R-r{)}^{2}}{24p}\right]}^{\frac{2}{3}}-{\left(\frac{R-r}{2}\right)}^{2}}$$ (36)

By substituting (29) into (24), the derivative for the deforming curve function of the electromagnetic diaphragm pump rubber diaphragm is computed as

$$f^{\prime }(x)=-\frac{x-\frac{R+r}{2}}{\sqrt{{\left(\frac{tE(R-r{)}^{2}}{24p}\right)}^{\frac{2}{3}}-{\left(x-\frac{R+r}{2}\right)}^{2}}}$$ (37)

## Section 4: Analysis on effective displacement of electromagnetic diaphragm pump

### Calculation of theoretical displacement for electromagnetic diaphragm pump

Suppose that the stroke for the center of the electromagnetic diaphragm pump rubber diaphragm is ±*h*. If the parasitical volume of the electromagnetic diaphragm pump is not considered, the theoretical displacement of the electromagnetic diaphragm pump is equal to twice the difference between the volume for big cone with the radius of *R* and the volume for small cone with the radius of *r*. The height of the big cone is computed as

$$h_{r}=\frac{rh}{\left(R-r\right)}$$ (38)

Furthermore, the height of the small cone is described by

$$h_{R}=\frac{Rh}{\left(R-r\right)}$$ (39)

Then the theoretical displacement of the electromagnetic diaphragm pump can be determined by taking advantage of (38) and (39).


$$V_{1}=2({\frac{\pi }{3}R}^{2}h_{R}-\frac{\pi }{3}r^{2}h_{r})=\frac{2\pi h(R^{2}+Rr+r^{2})}{3}$$ (40)

### Calculation of parasitical volume for electromagnetic diaphragm pump

By taking advantage of (36), the derivative for the parasitical volume of the electromagnetic diaphragm pump is expressed as

$${dV}_{2}=2\pi xf(x)dx$$ (41)

The elastic deformation of the electromagnetic diaphragm pump rubber diaphragm produces the parasitical volume under the action of fluid pressure, which will counteract part of the theoretical displacement and reduce effective displacement of the electromagnetic diaphragm pump. When the electromagnetic coil is energized, the pressure of the working cavity increases, and the elastic deformation of the electromagnetic diaphragm pump rubber diaphragm absorbs part of fluid, and the effective displacement is smaller than the theoretical displacement. When the electromagnetic coil is not electrified, the pressure of the working cavity decreases, and the electromagnetic diaphragm pump rubber diaphragm restores and releases part of fluid, and the effective suction volume of fluid is less than the theoretical displacement. The parasitical volume of the electromagnetic diaphragm pump is expressed as

$$V_{2}=\int_{r}^{R}2\pi xf(x)dx=\frac{\pi }{4}(R+r)\left\{{\left[\frac{tE(R-r{)}^{2}}{3p}\right]}^{\frac{2}{3}}arcsin{\left[\frac{3p(R-r)}{tE}\right]}^{\frac{1}{3}}-(R-r)\sqrt{{\left[\frac{tE(R-r{)}^{2}}{3p}\right]}^{\frac{2}{3}}-{\left(R-r\right)}^{2}}\right\}$$ (42)

### Calculation of effective displacement for electromagnetic diaphragm pump

The effective displacement of the electromagnetic diaphragm pump is equal to the difference between the theoretical displacement and the parasitical volume of the electromagnetic diaphragm pump, which is presented by

$$V=V_{1}-V_{2}=\frac{2\pi h(R^{2}+Rr+r^{2})}{3}-\frac{\pi }{4}(R+r)\left\{{\left[\frac{tE(R-r{)}^{2}}{3p}\right]}^{\frac{2}{3}}arcsin{\left[\frac{3p(R-r)}{tE}\right]}^{\frac{1}{3}}-(R-r)\sqrt{{\left[\frac{tE(R-r{)}^{2}}{3p}\right]}^{\frac{2}{3}}-{\left(R-r\right)}^{2}}\right\}$$ (43)

The volumetric efficiency of the electromagnetic diaphragm pump is equal to the ratio of the effective displacement to the theoretical displacement, namely:

$$\eta_{v}=\frac{V}{V_{1}}$$ (44)

### Section 5: Experimental results

In order to verify the results of the theoretical calculation, an experimental equipment was developed and a prototype electromagnetic diaphragm pump rubber diaphragm were designed and manufactured. The prototype electromagnetic diaphragm pump rubber diaphragm is made of HNBR material, and the parameters of the prototype electromagnetic diaphragm pump rubber diaphragm are depicted in Table 2.

**Table 2:** The parameters of the prototype electromagnetic diaphragm pump rubber diaphragm.

Parameter	Value
Shore hardness	HA75
Elastic modulus, *E*	10.985 MPa
Poisson’s ratio	0.4987
Thickness, *t*	2 mm
Radius of the inner driving ring, *r*	6 mm
Radius of the outer fixing ring, *R*	10 mm

**Fig 3:**
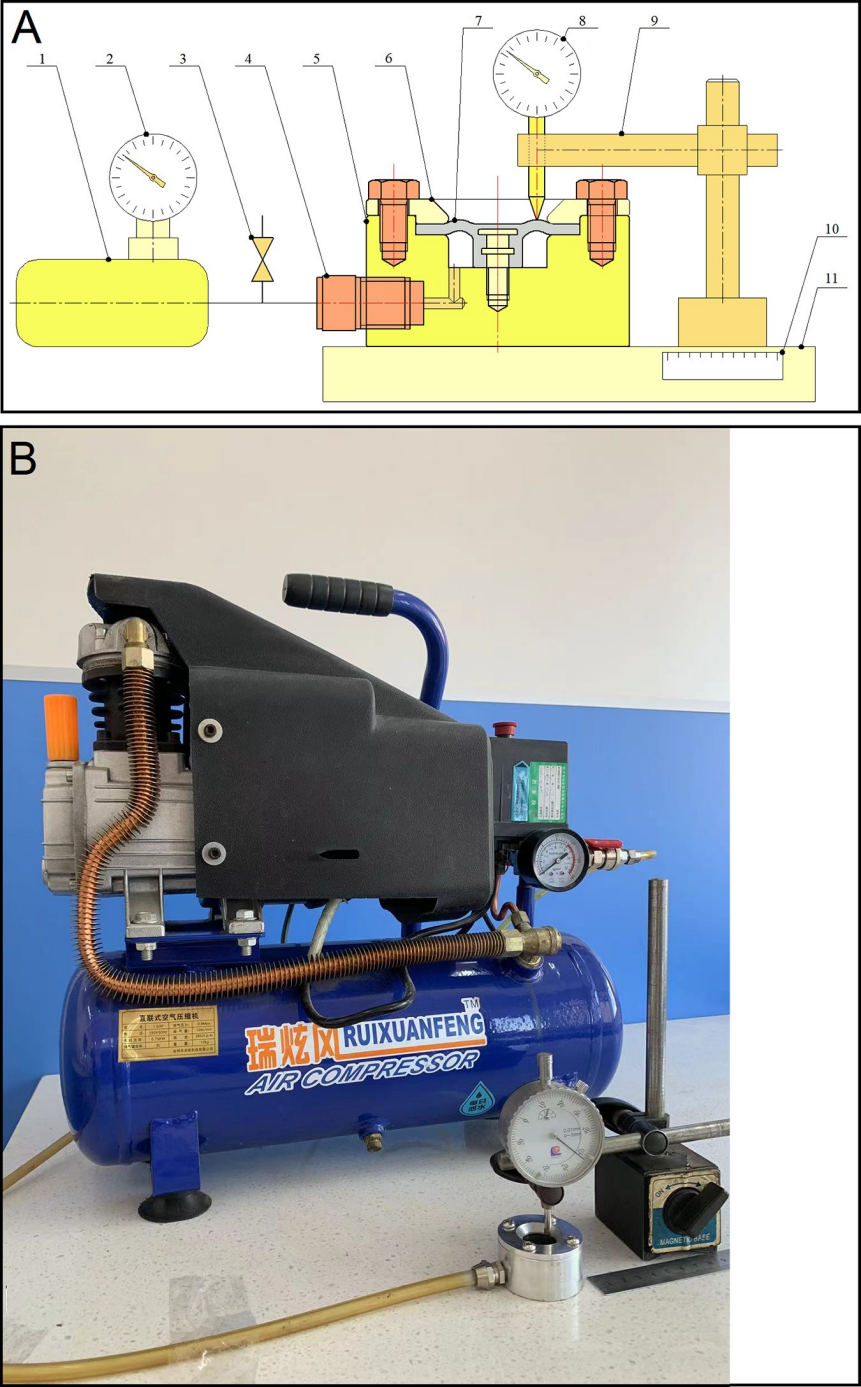
The experimental equipment (A) Structural scheme: 1-air compressor; 2-pressure gauge; 3-cut-off valve; 4-rubber hose joint; 5-pump body; 6-compression ring; 7-the prototype electromagnetic diaphragm pump rubber diaphragm; 8-dial indicator; 9-bracket; 10-ruler; 11-test plate (B) Photo.

The experimental equipment is mainly composed of air compressor, cut-off valve, rubber hose joint, the prototype electromagnetic diaphragm pump rubber diaphragm, pressure gauge, compression ring, pump body, dial indicator, ruler, test plate and bracket, as illustrated in Fig 3. The pump body and the prototype electromagnetic diaphragm pump rubber diaphragm make up a working cavity for containing air in.

The function of the air compressor is to supply compressed air to the working cavity for containing air in. The air compressor gas tank is used to maintain the pressure of air in the working cavity for containing air in. The function of the pressure gauge is to measure the output pressure of the air compressor. The cut-off valve is used to open and close the exhaust pipeline. Besides, the function of the dial indicator is to measure the axial deformation of the prototype electromagnetic diaphragm pump rubber diaphragm, and the ruler is used to test the radial displacement of the point for measuring the axial deformation. Table 3 illustrates the types and specifications of the experimental apparatus.

**Table 3:** Types and specifications of the experimental apparatus.

Apparatus	Types	Specifications	Value
Air compressor	1.0HP	Rated pressure	0.8 MPa
Pressure gauge	HONGQI	Range	0–1.6 MPa
Cut-off valve	J08H	Interface size	G1/8″
Dial indicator	0-5mm	Range	0–5 mm
		Accuracy	0.01 mm
Ruler	B100mm	Accuracy	0.5 mm

During the experiment, the cut-off valve is closed, the air compressor is switched on, and the dial indicator is installed on the bracket and can be moved onto the test plate, and the ruler is fixed along the radial direction of the prototype electromagnetic diaphragm pump rubber diaphragm on the test plate, and the pressure gauge is connected in parallel with the inlet pipe of the working cavity for containing air in, and the inner driving ring of the prototype electromagnetic diaphragm pump rubber diaphragm is installed in the center of the pump body by bolt, and the outer fixing ring of the prototype electromagnetic diaphragm pump rubber diaphragm is fixed on the pump body by the compression ring. The air compressor is switched off when the output pressure of the air compressor measured by the pressure gauge reaches 0.1, 0.2, 0.3, 0.4, 0.5 and 0.6 MPa, respectively. After that, the axial deformation of the prototype electromagnetic diaphragm pump rubber diaphragm is tested with the dial indicator.

**Fig 4:**
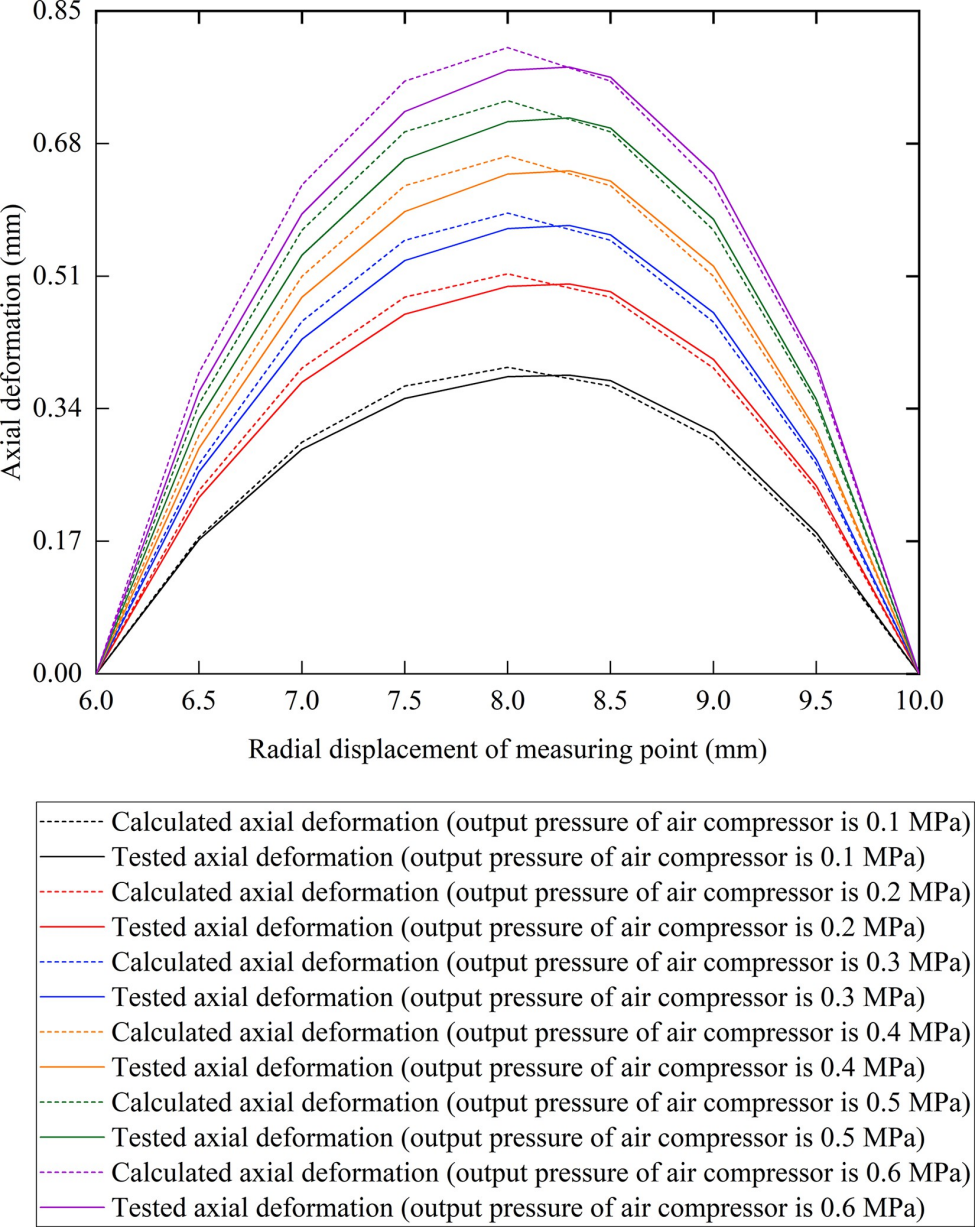
Comparison of axial deformation between the calculation results with the experimental results at different radial displacements of measuring point.

Fig 4 shows the axial deformations of the prototype electromagnetic diaphragm pump rubber diaphragm changing with the radial displacement of measuring point solution obtained by the theoretical calculation and by the experiment. It can be found in Fig 4 that both the tested axial deformation and the calculated axial deformation of the electromagnetic diaphragm pump rubber diaphragm increase by increasing the output pressure of the air compressor. Both the tested axial deformation and the calculated axial deformation of the electromagnetic diaphragm pump rubber diaphragm first increase and then decrease by increasing the radial displacement of the measuring point. Moreover, the tested axial deformation of the electromagnetic diaphragm pump rubber diaphragm is the maximum when the radial displacement of the measuring point is 8.3 mm, and the calculated axial deformation is the maximum when the radial displacement of the measuring point is 8 mm. This is because the area of the region surrounded by the inner driving ring of the electromagnetic diaphragm pump rubber diaphragm is smaller than the area of the region surrounded by the outer fixing ring of the electromagnetic diaphragm pump rubber diaphragm, and the turning point of the electromagnetic diaphragm pump rubber diaphragm moves outwards under the action of the output pressure for the air compressor. The largest difference between the tested axial deformation and the calculated axial deformation of the electromagnetic diaphragm pump rubber diaphragm is 0.04 mm, and the results of calculation show agreement with the experimental results.

## Section 6: Conclusion

In this study, a calculation method for deformation of the electromagnetic diaphragm pump rubber diaphragm is proposed. By establishing and analyzing the deformation model of the electromagnetic diaphragm pump rubber diaphragm, a theoretical relationship between the size of the electromagnetic diaphragm pump rubber diaphragm, the pressure of fluid and the deformation of the electromagnetic diaphragm pump rubber diaphragm, and the distribution of the deformation are determined. Experiment are carried out to verify the results of the theoretical analysis. The experimental results demonstrate that the biggest difference between the tested axial deformation and the calculated axial deformation of the electromagnetic diaphragm pump rubber diaphragm is 0.04 mm and the calculation results show agreement with the experimental results. The axial deformation of the electromagnetic diaphragm pump rubber diaphragm decreases by increasing the thickness of the electromagnetic diaphragm pump rubber diaphragm, and it will not be observed that the axial deformation of the electromagnetic diaphragm pump rubber diaphragm decreases linearly by increasing the thickness of the electromagnetic diaphragm pump rubber diaphragm. The calculation precision for the deformation of the electromagnetic diaphragm pump rubber diaphragm that bears high pressure of fluid can be further improved, which is the aim of our future study.

## Supporting information

S1 File Comparison of axial deformation between the calculation results with the experimental results at different radial displacements of measuring point. (XLSX)

S2 File Statistical significance test. (XLSX)

S3 File Mean and standard deviation. (XLSX)
